# Phylovar: toward scalable phylogeny-aware inference of single-nucleotide variations from single-cell DNA sequencing data

**DOI:** 10.1093/bioinformatics/btac254

**Published:** 2022-06-27

**Authors:** Mohammadamin Edrisi, Monica V Valecha, Sunkara B V Chowdary, Sergio Robledo, Huw A Ogilvie, David Posada, Hamim Zafar, Luay Nakhleh

**Affiliations:** Department of Computer Science, Rice University, Houston, TX 77005, USA; CINBIO, Universidade de Vigo, Vigo 36310, Spain; Department of Computer Science & Engineering, Indian Institute of Technology Kanpur, Kanpur 208016, India; University of Houston, Houston, TX 77204, USA; Department of Computer Science, Rice University, Houston, TX 77005, USA; CINBIO, Universidade de Vigo, Vigo 36310, Spain; Galicia Sur Health Research Institute (IIS Galicia Sur), SERGAS-UVIGO, Vigo, Spain; Department of Biochemistry, Genetics, and Immunology, Universidade de Vigo, Vigo 36310, Spain; Department of Computer Science & Engineering, Indian Institute of Technology Kanpur, Kanpur 208016, India; Department of Biological Sciences & Bioengineering, Institute of Technology Kanpur, Kanpur 208016, India; Mehta Family Centre for Engineering in Medicine, Indian Institute of Technology Kanpur, Kanpur 208016, India; Department of Computer Science, Rice University, Houston, TX 77005, USA

## Abstract

**Motivation:**

Single-nucleotide variants (SNVs) are the most common variations in the human genome. Recently developed methods for SNV detection from single-cell DNA sequencing data, such as SCIΦ and scVILP, leverage the evolutionary history of the cells to overcome the technical errors associated with single-cell sequencing protocols. Despite being accurate, these methods are not scalable to the extensive genomic breadth of single-cell whole-genome (scWGS) and whole-exome sequencing (scWES) data.

**Results:**

Here, we report on a new scalable method, Phylovar, which extends the phylogeny-guided variant calling approach to sequencing datasets containing millions of loci. Through benchmarking on simulated datasets under different settings, we show that, Phylovar outperforms SCIΦ in terms of running time while being more accurate than Monovar (which is not phylogeny-aware) in terms of SNV detection. Furthermore, we applied Phylovar to two real biological datasets: an scWES triple-negative breast cancer data consisting of 32 cells and 3375 loci as well as an scWGS data of neuron cells from a normal human brain containing 16 cells and approximately 2.5 million loci. For the cancer data, Phylovar detected somatic SNVs with high or moderate functional impact that were also supported by bulk sequencing dataset and for the neuron dataset, Phylovar identified 5745 SNVs with non-synonymous effects some of which were associated with neurodegenerative diseases.

**Availability and implementation:**

Phylovar is implemented in Python and is publicly available at https://github.com/NakhlehLab/Phylovar.

## 1 Introduction

With the advent of the first single-cell sequencing (SCS) techniques (Navin *et al.*, 2011; Tang *et al.*, 2009), the fields of single-cell genomics, transcriptomics, proteomics and epigenetics have witnessed remarkable growth over the last decade. Single-cell sequencing technologies have impacted our understanding in different fields of biology including developmental biology, immunology, microbiology and cancer biology (Kashima *et al.*, 2020; Lim *et al.*, 2020; Navin, 2014; Tang *et al.*, 2019; Wang and Navin, 2015). Single-cell DNA sequencing (scDNAseq), as one of the SCS technologies, provides insights into the somatic evolutionary process by sequencing the genomic contents of a complex tissue at a single-cell resolution (Navin, 2014; Navin *et al.*, 2011). Preparing scDNAseq data requires a whole-genome amplification (WGA) process to amplify the DNA material of a single cell to suffice the amount of DNA needed for sequencing. (Kashima *et al.*, 2020; Zafar *et al.*, 2018). WGA technologies, such as multiple displacement amplification (MDA) (Dean *et al.*, 2002; Spits *et al.*, 2006) and multiple annealing and looping-based amplification cycles (MALBAC) (Zong *et al.*, 2012) can elevate the noise level in scDNAseq data. The scDNAseq technical errors include allelic dropout (ADO), false-positive (FP) errors, false-negative (FN) errors and non-uniform coverage (Navin, 2014; Zafar *et al.*, 2018). ADO refers to cases where only one of the two alleles in a heterozygous mutation is amplified, resulting in the loss of the mutated allele. FP artifacts can appear due to uneven amplification or at the early stages of the amplification when the original nucleotide is substituted randomly. The non-uniform coverage over different genomic loci may result in missing data due to zero or insufficient coverage. The scDNAseq-specific technical errors fueled the development of tools such as Monovar (Zafar *et al.*, 2016) and SCcaller (Dong *et al.*, 2017) for detecting single-nucleotide variations (SNVs) from scDNAseq data. Although Monovar and SCcaller account for uneven coverage and scDNAseq-specific errors, more recent methods, SCIΦ (Singer *et al.*, 2018) and scVILP (Edrisi *et al.*, 2019), showed further improvement in overcoming the scDNAseq-specific technical errors by simultaneously inferring the cells’ phylogeny and SNVs. SCIΦ uses a Markov chain Monte Carlo (MCMC) algorithm to sample the joint posterior distribution of SNVs and the phylogenetic tree of the single cells and reports the tree(s) with the best posterior probability and the corresponding genotypes. scVILP is formulated as an instance of Mixed Integer Linear Programming (MILP) and it aims to find maximum likelihood estimation (MLE) of the observed read counts given the underlying genotype matrix. Here, the MILP solver is restricted to proposing only the genotype matrices that satisfy three-gamete condition in order to maximize the likelihood function (see Estabrook *et al.*, 1976; Fernández-Baca, 2001; Gusfield, 1991, 1997; Meacham, 1983; Semple and Steel, 2003 for more details on work related to inference under the three-gamete condition).

Although ‘regularizing’ the mutation detection by using a tree as a guide is a promising direction (Edrisi *et al.*, 2019; Kuipers *et al.*, 2020; Markowska *et al.*, 2021; Singer *et al.*, 2018), applying SCIΦ and scVILP to datasets with large number of loci such as in Evrony *et al.* (2015) and Wang *et al.* (2014) is challenged by either very long running time or large memory consumption of the methods—the major issues in SCIΦ and scVILP, respectively. Indeed, scVILP runs out of memory on all of the datasets considered in our study here, except for the smallest ones, which is why we do not report on the performance of scVILP. To address this challenge, we developed Phylovar, a likelihood-based method for phylogeny-aware inference of SNVs from scDNAseq datasets consisting of a large number of loci. To simplify likelihood calculations for large-scale data, we assume that mutations occur following an infinite-sites assumption (ISA) (Deshwar *et al.*, 2015; Jahn *et al.*, 2016; Singer *et al.*, 2018). Using this model, Phylovar finds the tree topology and the placement of mutations on ancestral single cells that maximize the likelihood of the erroneous observed read counts given the genotypes. Utilizing a vectorized formulation for likelihood calculations, Phylovar benefits from the vectorized operations in matrix manipulation packages such as NumPy (Harris *et al.*, 2020) to scale up to many loci. We compared the SNV calling accuracy, memory consumption and running time of Phylovar against those of the existing methods, Monovar and SCIΦ, through a simulation study. We found that Phylovar outperforms SCIΦ in terms of running time with the same accuracy, while being more accurate than Monovar. Furthermore, we applied our method to two biological datasets: a triple-negative breast cancer (TNBC) dataset (Wang *et al.*, 2014) consisting of 32 single cells and 3375 candidate loci, as well as the dataset from Evrony *et al.* (2015) containing 16 normal human neuron cells and 2 489 545 candidate loci. For the TNBC data, Phylovar inferred 652 SNVs with ‘high’ or ‘moderate’ functional impact, out of which 550 (84%) were also supported by bulk sequencing. For the neuron cells, Phylovar identified 5745 SNVs with non-synonymous effects some of which were related to neurodegenerative diseases. To the best of our knowledge, Phylovar is the first scDNAseq SNV caller that can utilize the underlying tree structure even when the dataset contains millions of genomic loci.

## 2 Materials and methods

The input to Phylovar consists of the reference and variant count matrices, denoted by $R=(r_{ij})\in N_{0}{}^{N\times M}$ and $V=(v_{ij})\in N_{0}{}^{N\times M}$, where *N* and *M* represent the number of single cells and candidate loci, respectively. Each entry in **R** and **V** represents the number of reference and variant counts, respectively, at cell *i* and site *j*. These count matrices are obtained from an input file in mpileup format. Here, candidate loci are defined as the genomic loci with a significant number of variant reads. This significance is measured by a statistic test. Note that these loci may not necessarily contain SNVs since the variant reads might be artifacts of scDNAseq technical errors. In all experiments reported below, we used SCIΦ’s likelihood ratio test described in Singer *et al.* (2018) to identify candidate loci for the analyses. If the total read coverage at a cell and a candidate site is less than *λ*, the corresponding entry is treated as missing data. We used *λ *= 1 in practice.

### 2.1 Single-cell genotype error model

Our genotype model considers bi-allelic genotype with 0 and 1 representing the absence and presence of a mutation, respectively. We differentiate true genotypes from those being subject to scDNAseq errors that propagate from WGA to the sequencing library—called library genotypes. Let $G=(g_{ij})\in {\left\{0,1\right\}}^{N\times M}$ be the binary matrix containing the true genotypes where *g_ij_* represents the true genotype at cell *i* and locus *j*. Similarly, we denote the library genotype matrix by $\Psi =(\psi_{ij})\in {\left\{0,1\right\}}^{N\times M}$. We assume library preparation process introduces FP and FN errors into the data resulting in difference between true genotypes and their corresponding library genotypes. Let *α* and *β* denote the FP and FN error rates, respectively. Then, the probability of the library genotype given true genotype and error rates are given by the following error model adopted from SiFit (Zafar *et al.*, 2017) and SiCloneFit (Zafar *et al.*, 2019):

(1) $$P(\psi_{ij}|g_{ij},\alpha ,\beta )=\{\begin{array}{cc} \alpha & \text{if}\;\psi_{ij}=1,\;\text{and}\;g_{ij}=0\\ 1-\alpha & \text{if}\;\psi_{ij}=0,\;\text{and}\;g_{ij}=0\\ \beta & \text{if}\;\psi_{ij}=0,\;\text{and}\;g_{ij}=1\\ 1-\beta & \text{if}\;\psi_{ij}=1,\;\text{and}\;g_{ij}=1 \end{array}.$$

### 2.2 Single-cell read count model

For the convenience of notation, let $c_{ij}=r_{ij}+v_{ij}$ denote the total read coverage at cell *i* and locus *j*. We assume that variant read counts follow a binomial distribution whose success probability depends on the value of the library genotype:

(2) $$P\left(r_{ij},v_{ij}|\psi_{ij}\right)=\{\begin{array}{cc} \left(\begin{array}{c} c_{ij}\\ v_{ij} \end{array}\right)\mu_{0}{}^{v_{ij}}{\left(1-\mu_{0}\right)}^{r_{ij}} & \text{if}\;\psi_{ij}=0\\ \left(\begin{array}{c} c_{ij}\\ v_{ij} \end{array}\right)\mu_{1}{}^{v_{ij}}{\left(1-\mu_{1}\right)}^{r_{ij}} & \text{if}\;\psi_{ij}=1 \end{array}.$$

The variables *μ*_0_ and *μ*_1_ are the success probabilities associated with reference and alternate alleles, respectively. In practice, we set *μ*_0_ to 0.001, which is at the same order of magnitude for the error rate in different Illumina sequencing platforms (Stoler and Nekrutenko, 2021). We used 0.5 for the value of *μ*_1_, which is the mean of variant read counts for a heterozygous mutation.

### 2.3 Tree model

Our tree model consists of two components: a binary tree topology T=(V,E)—where *V* denotes the set of nodes, and *E* is the set of edges—and a mutation placement for each genomic locus *j*, $M_{j}\in {\left\{0,1\right\}}^{2N-1}$. The latter is a binary vector of length 2N−1 containing binary elements for each leaf or internal node in *V*. We take $M_{j}[q]=1$ to denote that a mutation occurred at node *q* during the evolutionary history of locus *j*. In our model, we assume mutations evolve following the ISA. According to this model, at most one element in $M_{j}$ is allowed to be 1. This vector requires each node to have an index from {1,…,2N−1}. For simplicity, we map indices {1,…,N} to the leaves/single cells and use the same mapping for the single cells in all tree topologies.

### 2.4 Log-likelihood function

Assuming independence across sites/loci, the log-likelihood function of read counts given true genotypes **G**, error rates (α,β), underlying tree topology *T* and mutation placements for all loci (denoted by M) are:

(3) $$\begin{array}{cc} & F(R,V|G,T,M,\alpha ,\beta )=F(R,V|G,\alpha ,\beta )\\ & =\sum_{i=1}^{N}\sum_{j=1}^{M}\;\text{log}\;\left\{\sum_{\psi }P\left(r_{ij},v_{ij}|\psi_{ij}\right)P(\psi_{ij}|g_{ij},\alpha ,\beta )\right\}. \end{array}$$

Note that the above likelihood is based on **G** rather than *T* and M directly, as **G** is derived from *T* and M. Therefore, we can drop *T* and M from Equation (3). It can be shown that after marginalizing out *ψ*’s, $\text{log}\;P(r_{ij},v_{ij}|g_{ij},\alpha ,\beta )=\text{log}\;\{\sum_{\psi }P\left(r_{ij},v_{ij}|\psi_{ij}\right)P(\psi_{ij}|g_{ij},\alpha ,\beta )\}$ can be simplified as follows:

(4) $$\begin{array}{cc} & \;\text{log}\;P(r_{ij},v_{ij}|g_{ij},\alpha ,\beta )=\text{log}\;\left(\begin{array}{c} c_{ij}\\ v_{ij} \end{array}\right)\\ & +(1-g_{ij})\;\text{log}\;\{\mu_{0}{}^{v_{ij}}{\left(1-\mu_{0}\right)}^{r_{ij}}(1-\alpha )+\mu_{1}{}^{v_{ij}}{\left(1-\mu_{1}\right)}^{r_{ij}}\alpha \}\\ & +g_{ij}\;\text{log}\;\{\mu_{0}{}^{v_{ij}}{\left(1-\mu_{0}\right)}^{r_{ij}}\beta +\mu_{1}{}^{v_{ij}}{\left(1-\mu_{1}\right)}^{r_{ij}}(1-\beta )\}. \end{array}$$

Here, the log-likelihood values of the missing data are assumed to be 0. The MLE solution is obtained as

(5) $$(G^{*},T^{*},M^{*},\alpha^{*},\beta^{*})=\underset{G,T,M,\alpha ,\beta }{\mathrm{argmax}}\left\{F(R,V|G,T,M,\alpha ,\beta )\right\}.$$

### 2.5 Hill-climbing search algorithm

Phylovar infers the underlying phylogeny of single cells and their genotypes simultaneously in a hill-climbing fashion. At each step, the log-likelihood function is evaluated and updated by proposing one of the underlying parameters including the tree, mutation placements and error rates. We start the search by reconstructing an initial tree topology. To obtain this tree, first, we create the matrix of initial genotypes, $G^{\left(0\right)}$, as follows:

(6) $$g_{ij}^{\left(0\right)}=\{\begin{array}{ccc} 1 & \text{if}\;\; & \;\text{log}\;P(r_{ij},v_{ij}|g_{ij},\alpha^{\left(1\right)},\beta^{\left(1\right)}){|}_{\begin{array}{c} g_{ij}=1\\ \alpha^{\left(1\right)}=0\\ \beta^{\left(1\right)}=0 \end{array}}>\\ & & \;\text{log}\;P(r_{ij},v_{ij}|g_{ij},\alpha^{\left(1\right)},\beta^{\left(1\right)}){|}_{\begin{array}{c} g_{ij}=0\\ \alpha^{\left(1\right)}=0\\ \beta^{\left(1\right)}=0 \end{array}}\\ 0 & \text{Otherwise} & \end{array}.$$

Here, $\left(\alpha^{\left(1\right)},\beta^{\left(1\right)})=(0,0\right)$ are the initial estimates of the error rates. Given $G^{\left(0\right)}$, we calculate the pairwise Hamming distances between the single cells and build an initial tree topology, $T^{\left(1\right)}$, using the neighbor-joining algorithm (Saitou and Nei, 1987). Given the proposed parameters $\left(T^{\left(1\right)},\alpha^{\left(1\right)},\beta^{\left(1\right)}\right)$, the mutation placement with highest log-likelihood for each site *j*—denoted by $M_{j}^{*(1)}$—is determined (see below for more details) yielding the genotype matrix at first iteration, $G^{\left(1\right)}$ and the first log-likelihood value $F(R,V|G^{\left(1\right)},T^{\left(1\right)},M^{*(1)},\alpha^{\left(1\right)},\beta^{\left(1\right)})$. At each iteration t≥2, either new error rates are estimated (see below for more details) or a new tree is proposed by performing tree rearrangement techniques including *subtree pruning and re-grafting* (SPR), *nearest-neighbor interchange* (NNI) and swapping two random leaves. The proposed parameters are accepted if the new log-likelihood value is greater than or equal to the log-likelihood in the previous iteration. In case of stochastic hill-climbing, the acceptance probability of the newly proposed log-likelihood value is:

(7) $$\min\{1,\frac{F(R,V|G^{\left(t\right)},T^{\left(t\right)},M^{*(t)},\alpha^{\left(t\right)},\beta^{\left(t\right)})}{F(R,V|G^{\left(t-1\right)},T^{\left(t-1\right)},M^{*(t-1)},\alpha^{\left(t-1\right)},\beta^{\left(t-1\right)})}\}.$$

The search procedure terminates when the log-likelihood does not improve after a user-specified number of iterations or when it reaches the maximum number of iterations.

### 2.6 Proposing new error rates

The new error rates at iteration *t* are calculated using the following equations from the entries of $G^{\left(t-1\right)}$ and $G^{\left(0\right)}$:

(8) $$\alpha^{\left(t\right)}=\frac{\sum_{i,j}[g_{ij}^{\left(0\right)}=1\wedge g_{ij}^{\left(t-1\right)}=0\wedge c_{ij}\neq 0]}{\sum_{i,j}[c_{ij}\neq 0]},$$

 

(9) $$\beta^{\left(t\right)}=\frac{\sum_{i,j}[g_{ij}^{\left(0\right)}=0\wedge g_{ij}^{\left(t-1\right)}=1\wedge c_{ij}\neq 0]}{\sum_{i,j}[c_{ij}\neq 0]}.$$

Here, the number of 0 entries in $G^{\left(0\right)}$ that were ‘corrected’ to 1 in $G^{\left(t-1\right)}$ provides a measure of what a more realistic *α* would be through the hill-climbing trajectory. The same rationale applies to proposing a new value of *β*. In Equations (8) and (9), $\alpha^{\left(t\right)}$ and $\beta^{\left(t\right)}$ indicate the new values of *α* and *β* at iteration *t*, respectively. Here, $g_{ij}^{\left(0\right)}$ and $g_{ij}^{\left(t-1\right)}$ denote the entries of the initial genotype matrix and the genotype matrix at iteration *t–*1, respectively. The term $c_{ij}\neq 0$ indicates the entries with non-zero read counts. The symbol ∧ represents the logical AND operator.

### 2.7 Finding the best mutation placement

Given a topology $T^{\left(t\right)}$ and a site *j*, each possible mutation placement on $T^{\left(t\right)}$ yields a unique genotype configuration at the level of single cells. We seek the mutation placement with the highest log-likelihood. Let $M^{\left(t\right)}{\left(v_{k}\right)}_{k\in \{1,\ldots ,2N-1\}}$ denote the mutation placement when the *k*^th^ node is mutated. As a special case, let $M^{\left(t\right)}(v_{2N})$ represent the absence of mutation. Because the set of all possible mutation placements is the same for all the sites, we dropped the index *j* from these two notations. To summarize the effect of all possible ISA mutation placements on genotype configurations, we define $S^{\left(t\right)}=(s_{ki}^{\left(t\right)})\in {\left\{0,1\right\}}^{2N\times N}$ whose *k*^th^ row, $S_{k*}^{\left(t\right)}$, represents the genotype configuration corresponding to $M^{\left(t\right)}(v_{k})$. We formally define the mapping from mutation placements to genotypes, denoted by Φ, as follows:

(10) $$\Phi \left(M^{\left(t\right)}(v_{k})\right)=S_{k*}^{\left(t\right)}=[s_{k1}^{\left(t\right)},\ldots ,s_{kN}^{\left(t\right)}],\;$$

 

(11) $$s_{ki}^{\left(t\right)}=\{\begin{array}{cc} 1 & \text{if}\;v_{i}\in T_{v_{k}}^{\left(t\right)}\\ 0 & \text{if}\;v_{i}\notin T_{v_{k}}^{\left(t\right)}\;\text{or}\;k=2N \end{array}.$$

where i∈{1,…,N} and k∈{1,…,2N}. Here, $T_{v_{k}}^{\left(t\right)}$ denotes the subtree rooted at node *v_k_*. Note that the mapping Φ is one-to-one, so we can use its inverse to retrieve the genotypes given a mutation placement. In addition to $S^{\left(t\right)}$, we define two other matrices that store the log-likelihood values from Equation (4), one assuming all genotypes are 0, called matrix of zero-allele likelihoods, $Z^{\left(t\right)}$ and the other assuming all genotypes are 1, called matrix of one-allele likelihoods, $O^{\left(t\right)}$. Formally, we define the matrices $O^{\left(t\right)}=(o_{ij}^{\left(t\right)})\in {\left(-\infty ,0\right]}^{N\times M}$ and $Z^{\left(t\right)}=(\zeta_{ij}^{\left(t\right)})\in {\left(-\infty ,0\right]}^{N\times M}$ as follows:

(12) $$o_{ij}^{\left(t\right)}=\text{log}\;P(r_{ij},v_{ij}|g_{ij},\alpha^{\left(t\right)},\beta^{\left(t\right)}){|}_{g_{ij}=1},$$

 

(13) $$\zeta_{ij}^{\left(t\right)}=\text{log}\;P(r_{ij},v_{ij}|g_{ij},\alpha^{\left(t\right)},\beta^{\left(t\right)}){|}_{g_{ij}=0}.$$

It can be shown that the following matrix multiplication results in a matrix $X^{\left(t\right)}=(\chi_{kj}^{\left(t\right)})\in {\left(-\infty ,0\right]}^{2N\times M}$ whose each element $\chi_{kj}^{\left(t\right)}$ is equal to the log-likelihood value of $M^{\left(t\right)}(v_{k})$ at site *j*:

(14) $$X^{\left(t\right)}=S^{\left(t\right)}O^{\left(t\right)}+(J_{2N,N}-S^{\left(t\right)})Z^{\left(t\right)}.$$

Here, $J_{2N,N}$ is matrix of all-ones. The best mutation placement at site *j*, $M_{j}^{*(t)}$, is associated with the highest value in the *j*^th^ column of $X^{\left(t\right)}$:

(15) $$M_{j}^{*(t)}=M^{\left(t\right)}(v_{k^{*}}).$$

The index corresponding to the highest value is denoted by $k^{*}$:

(16) $$k^{*}=\underset{k\in \{1,\ldots ,2N\}}{\mathrm{argmax}}\left\{\chi_{kj}^{\left(t\right)}\right\}.$$

Using $\Phi^{-1}$, we can determine the best genotype configuration at site *j* which constitutes the $j^{\text{th}}$ column of $G^{\left(t\right)}$ using

(17) $$G_{*j}^{\left(t\right)}=S_{k^{*}\;*}^{\left(t\right)}=\Phi^{-1}\left(M^{\left(t\right)}(v_{k^{*}})\right).$$

Finally, $G^{\left(t\right)}$ is the concatenation of best genotypes configurations at all sites:

(18) $$G^{\left(t\right)}=[G_{*1}^{\left(t\right)},\ldots ,G_{*M}^{\left(t\right)}].$$

## 3 Results and discussion

### 3.1 Simulation study

We first compared the computational efficiency and SNV calling accuracy of Phylovar, SCIΦ and Monovar using synthetic datasets simulated under five scenarios: (i) varying the number of mutations, (ii) varying the number of cells, (iii) varying the ADO rates, (iv) copy number effect and (v) violation of ISA. We simulated the datasets using the simulator introduced in Singer *et al.* (2018). In the first scenario, we investigated how Phylovar performs compared to the other methods when increasing the number of mutations dramatically to a large extent.

**Fig. 1.**
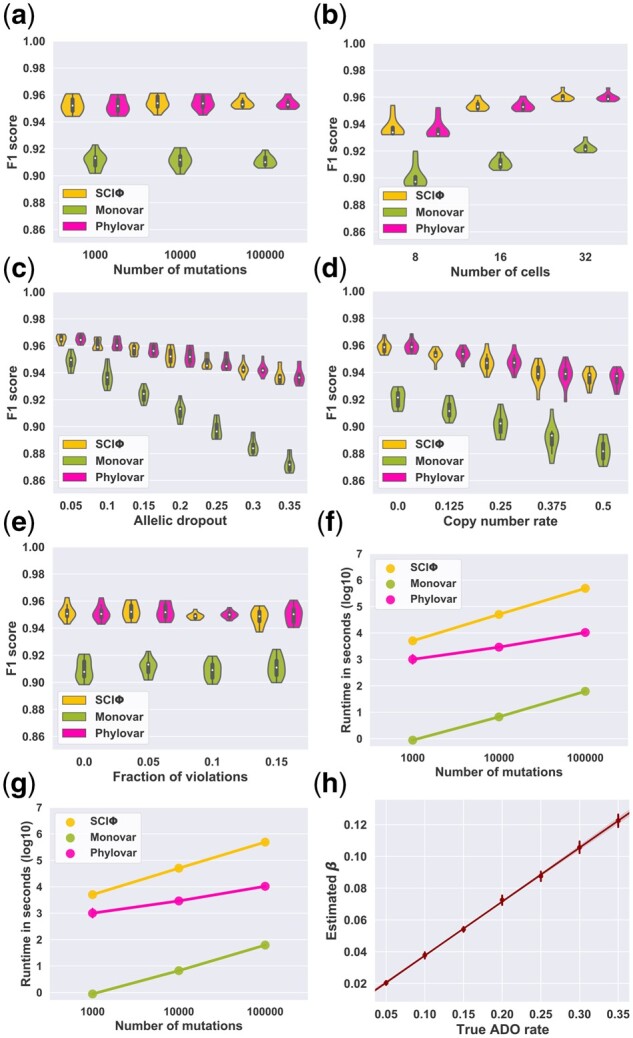
Summary statistics of different benchmarking experiments. (**a–e**) F1 accuracy of the methods from simulated data with different number of mutations (a), number of cells (b), ADO rate (c), copy number rate (d) and fraction of ISA violations (e). (**f, g**) Runtime of the methods on simulated data with varying number of mutations (f) and varying number of cells (g). (**h**) Linear regression between estimated false-negative error rates (*β*’s) and actual ADO rates used for simulated data

We simulated datasets containing 16 single cells with 1000, 10^4^ and 10^5^ mutations. For each mutation value, ten datasets were generated. Phylovar’s accuracy was comparable to that of SCIΦ in terms of F1 measure, while both SCIΦ and Phylovar were more accurate than Monovar because of accounting for evolutionary history (Fig. 1a). To measure the running time of each method, we used the CPU clock time (in seconds and log scale) during its execution. Figure 1f shows that the running time of each method increased with the number of mutations. For the largest dataset with 10^5^ mutations, Phylovar was approximately two orders of magnitude faster than SCIΦ.

In the second scenario, we sought to answer how the methods’ performances depend on the number of cells. Here, we fixed the number of mutations at 10^5^ and varied the number of cells, N∈{8,16,32}. For each setting, we generated ten datasets. The F1 accuracy scores of all methods improved as the number of cells increased (Fig. 1b). Again, Phylovar’s accuracy was comparable to that of SCIΦ while it outperformed Monovar. We observed that increasing the number of single cells improved the accuracy of all methods more than increasing the number of mutations. As demonstrated in Figure 1g, similar to the first scenario, the running time of Phylovar was almost two orders of magnitude less than that of SCIΦ.

In the third scenario, the ADO rate was varied while cells and mutations were fixed at 16 and 1000. We selected ADO rates from the values {0,0.05,0.1,0.15,0.2,0.25,0.3,0.35}, and generated ten datasets for each ADO value. Figure 1c shows that both SCIΦ and Phylovar were more robust to high ADO rates than Monovar due to the utilization of the underlying single-cell phylogeny.

Since Phylovar can estimate the false-negative error rates (denoted by *β*), we measured the correlation between the ADO rates used for generating the simulated datasets and the estimated *β*’s. As demonstrated in Figure 1h, these two values were highly correlated (the Pearson correlation coefficient was 0.991). It is worth noting that based on the linear regression line, the estimated *β* was almost half of the true ADO, pointing to the difference between the dropout mechanism in the simulator and our definition of *β* (see Section 2). Given an ADO rate *μ*, the simulator chooses *μ* fraction of the mutations. It changes $\frac{\mu }{2}$ of them into reference genotype, and $\frac{\mu }{2}$ of them into homozygous mutations; the *β* in our model indicates the probability of a mutation becoming reference genotype implying $\beta \approx \frac{\mu }{2}$, which we can observe in Figure 1h.

Since Phylovar assumes the read counts are originated from diploid strands, in the fourth scenario, additional wild-type alleles were introduced to the read counts to imitate the effect of copy number changes. The simulator randomly selects a fraction of mutated loci (named copy number rate), and chooses *c* extra copies for each loci with probability $\frac{1}{2^{c}}$ (Singer *et al.*, 2018). We increased the copy number rate from 0 to 0.5 with step size 0.125. For each value, we generated ten datasets containing 16 cells and 1000 mutations. Figure 1d shows that the SNV calling accuracy of the methods decreased as more mutated loci were subject to copy number changes.

In the fifth scenario, we were interested in observing how violations of ISA affect the SNV calling accuracy of the methods. Given a fraction of mutations, the simulator randomly selects half of them to recur in different branches, and the rest of them to be lost in the same subtree. We increased the fraction of mutations subject to ISA violations from 0 to 0.15 with 0.05 step size. For each value we generated ten datasets with 16 cells and 1000 mutations. Figure 1e shows that all three methods had a stable performance as the fraction of ISA violations increased. This observation suggests that even though the phylogenies inferred by SCIΦ and Phylovar might be inaccurate due to the presence of violations of their evolutionary model, the effect of such violations on mutation inference is negligible.

### 3.2 Application to real data

**Fig. 2.**
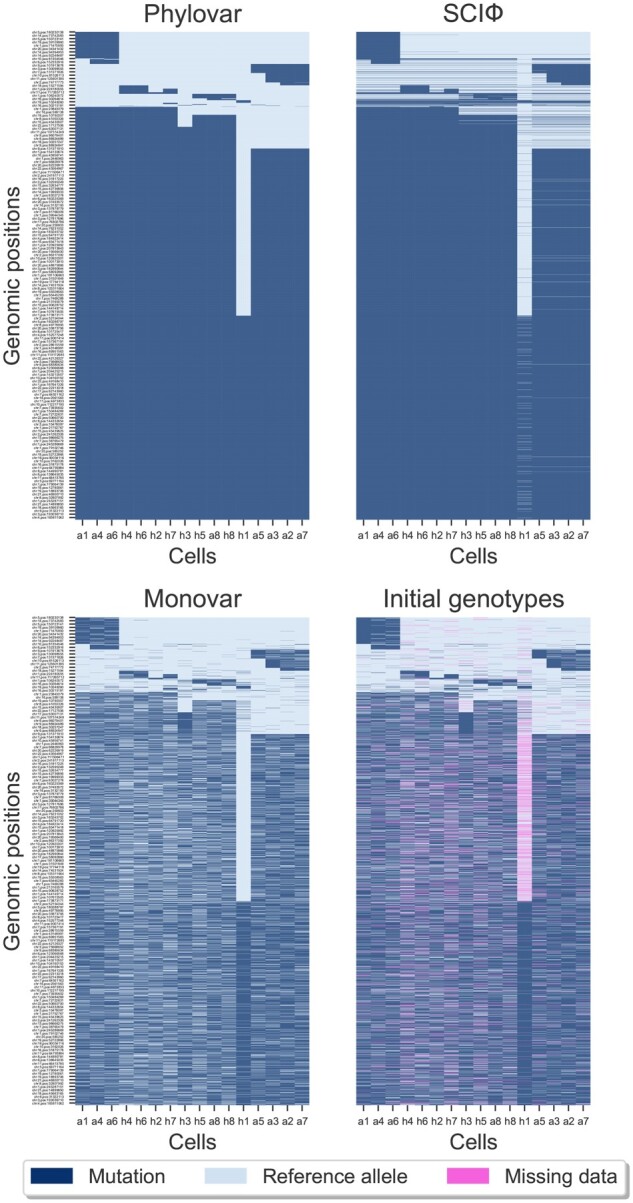
Clustered heatmaps of mutation calls by different methods performed on the TNBC dataset. Here, rows and columns represent the genomic loci and the single cells, respectively. The pixels show mutation calls (dark blue), reference alleles (light blue) and missing data (pink). The initial genotypes are the initial estimates of genotypes considering no error rates and no underlying phylogeny at the starting step of Phylovar’s search algorithm (A color version of this figure appears in the online version of this article)

We applied Phylovar on two human scDNAseq datasets. The first dataset consists of single-cell whole-exome sequencing (scWES) samples from a triple-negative breast cancer (TNBC) patient (Wang *et al.*, 2014). Since the population sequencing data from bulk tumor and matched normal tissue are available for the TNBC dataset, the number of mutations shared by scWES and bulk data provides us a metric for measuring the accuracy of our approach. The TNBC dataset consists of 16 diploid cells, eight hyperdiploid/aneuploid cells and eight hypodiploid cells (Wang *et al.*, 2014). Given the control normal cells, SCIΦ’s likelihood ratio test identifies the loci likely to contain somatic mutations. Applying this statistic test on the input mpileup file resulted in 3375 candidate loci on which we applied Phylovar. Phylovar was run with ten parallel hill-climbing chains, each for 100 000 iterations on a pool of five CPU’s, each with 48 cores (AMD EPYC 7642) on a node with 192 GB RAM. The total runtime was 91 min. Phylovar inferred an 18.21% false-negative error rate and a 1.03% false-positive error rate from TNBC data. We ran SCIΦ and Monovar with default parameters; SCIΦ and Monovar terminated after 10 h and 144 min, respectively. Figure 2 shows the three methods’ mutation calls on TNBC data from the overlapping sites as well as the initial genotype matrix at the first iteration of our hill-climbing search. We performed hierarchical clustering with Ward’s minimum variance method implemented in Python’s SciPy package (Virtanen *et al.*, 2020) on the genotype matrix for better visualization. We observed concordance between the calls from Phylovar and SCIΦ while Monovar’s calls are noisy and resemble Phylovar’s initial genotypes.

**Fig. 3.**
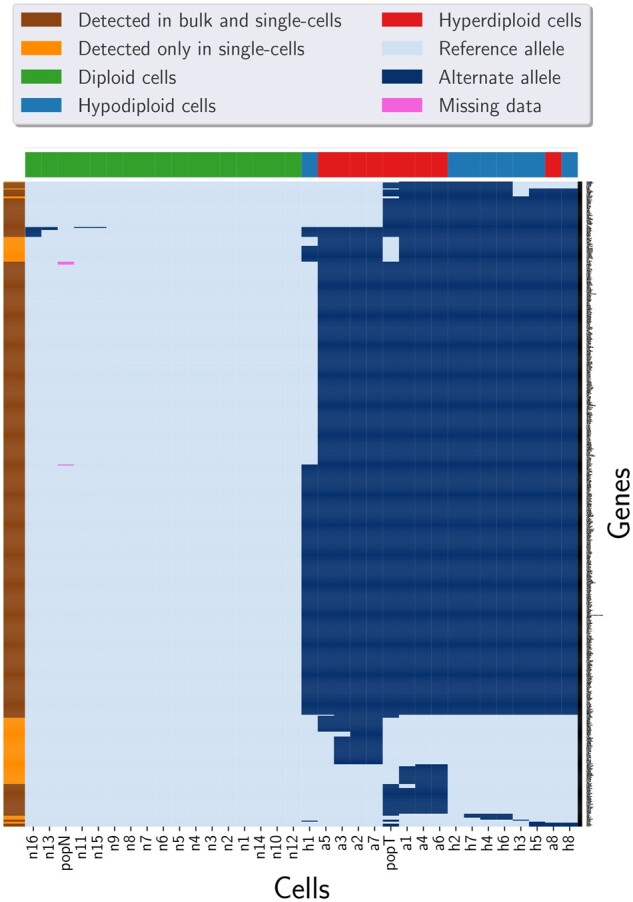
Clustered heatmap of the mutations detected by Phylovar from TNBC data and population sequencing data of tumor and matched normal tissues. The pixels show mutation calls (dark blue), reference alleles (light blue) and missing data (pink). Columns (cells) are colored according to the ploidy of the cells. The colors of the rows (genes) indicate whether the SNV was found in bulk data or not. Here, popT and popN are the tumor and normal population sequencing samples, respectively. Out of 3375 candidate loci, 652 loci contained SNVs with high or moderate functional effects in the single-cell data among which 550 mutations were found in bulk data as well, which is 84% of the single-cell calls (A color version of this figure appears in the online version of this article)

**Fig. 4.**
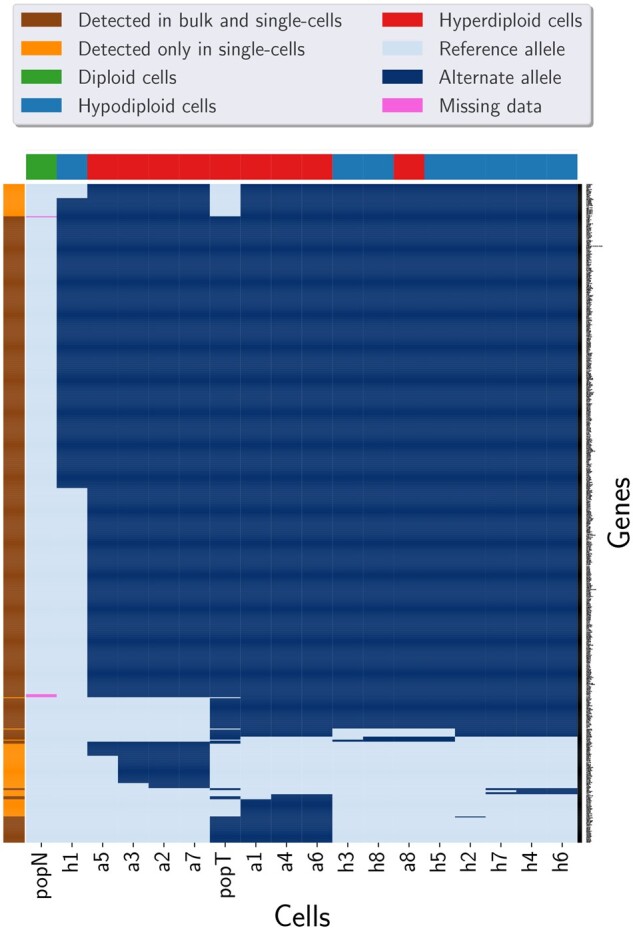
Clustered heatmap of the mutations detected by SCIΦ from TNBC data and population sequencing data of tumor and matched normal tissues. The pixels show mutation calls (dark blue), reference alleles (light blue) and missing data (pink). Columns (cells) are colored according to the ploidy of the cells. Since SCIΦ discarded the diploid cells from the final output, only hypodiploid and hyperdiploid cells are demonstrated here. The colors of the rows (genes) indicate whether the SNV was found in bulk data or not. Here, popT and popN are the tumor and normal population sequencing samples, respectively. SCIΦ detected 652 SNVs with high or moderate functional effects in the single-cell data among which 550 mutations were found in bulk data as well, which is 84% of the single-cell calls (A color version of this figure appears in the online version of this article)

**Fig. 5.**
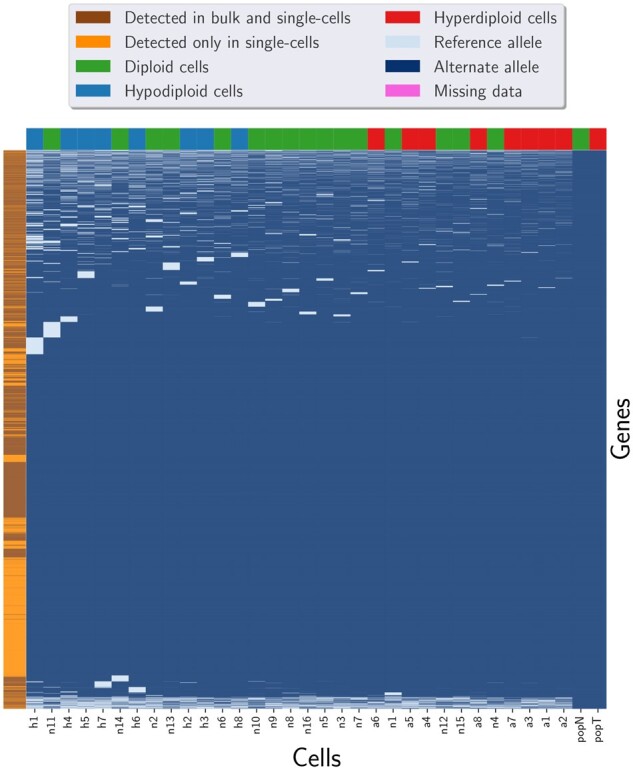
Clustered heatmap of the mutations detected by Monovar from TNBC data and population sequencing data of tumor and matched normal tissues. The pixels show mutation calls (dark blue), reference alleles (light blue) and missing data (pink). Columns (cells) are colored according to the ploidy of the cells. The colors of the rows (genes) indicate whether the SNV was found in bulk data or not. Here, popT and popN are the tumor and normal population sequencing samples, respectively. Monovar detected 18 187 high or moderate non-synonymous mutations in the single-cell data from which 10 981 mutations were found in the bulk data as well which is 60% of the single-cell calls (A color version of this figure appears in the online version of this article)

To annotate the mutations, we applied snpEff (Cingolani *et al.*, 2012) on the SNVs detected by Phylovar. Out of 3375 candidate loci, 652 loci contained SNVs with ‘high’ or ‘moderate’ functional effects (see https://pcingola.github.io/SnpEff/se_inputoutput/ for details on the types of variants’ effects and their descriptions). Then, we ran HaplotypeCaller (GATK version 4.2.0.0) for mutation calling on the bulk tumor and normal samples. Among 652 SNVs in single cells, 550 (84%) mutations were found in bulk data (Fig. 3). Performing this analysis on the results of SCIΦ yielded the same results as for Phylovar: 652 loci contained SNVs with high or moderate functional effect, out of which 550 mutations overlapped with the calls from bulk data. Monovar detected 18 187 high or moderate non-synonymous mutations in the single-cell data from which 10 981 mutations were found in the bulk data as well which is 60% of the single-cell calls (Figs. 4 and 5).

**Fig. 6.**
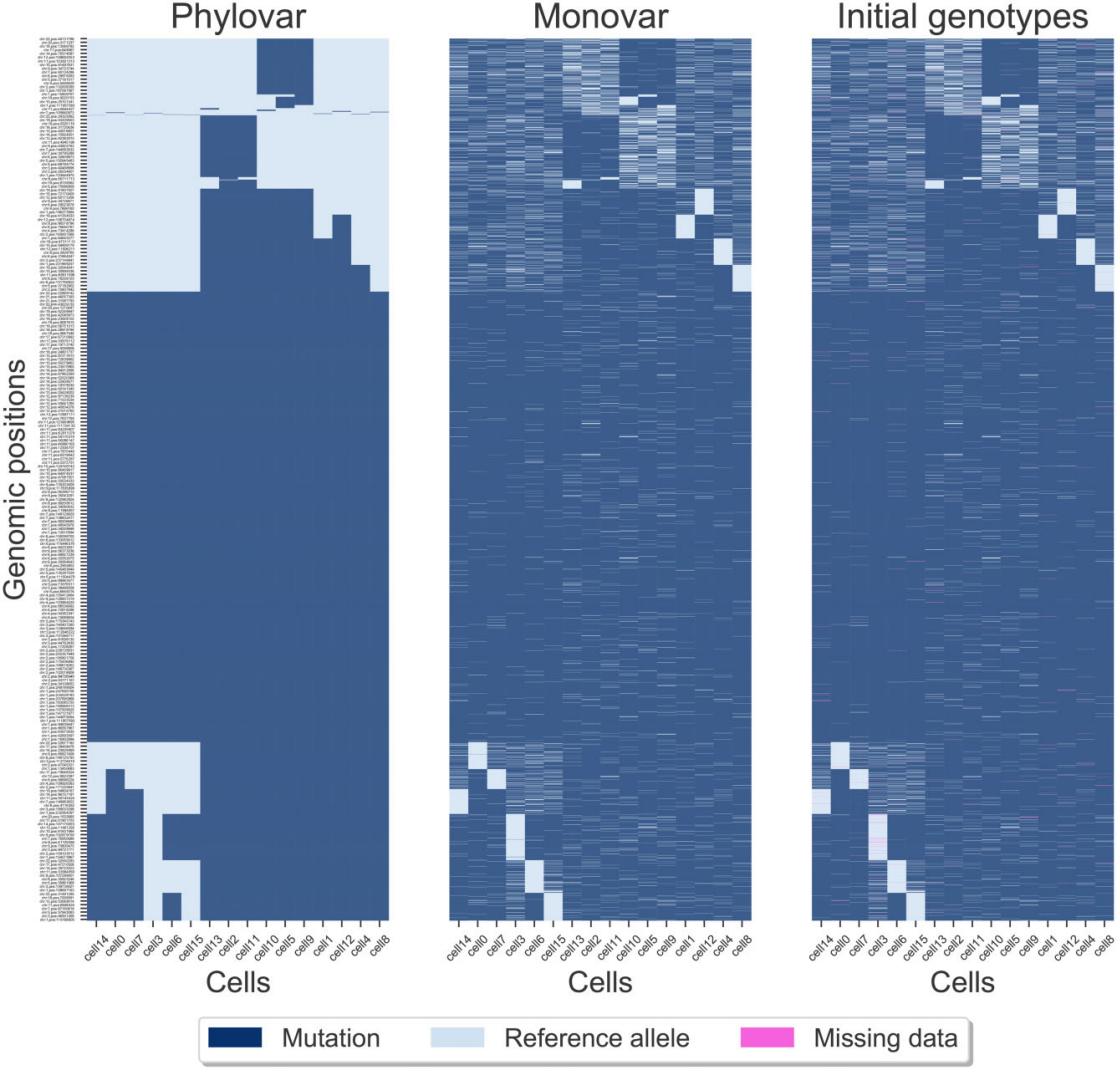
Clustered heatmaps of mutation calls by different methods performed on neuron cells. Rows and columns represent the genomic loci and the single cells, respectively. The pixels show mutation calls (dark blue), reference alleles (light blue) and missing data (pink). The initial genotypes are the initial estimates of genotypes considering no error rates and no underlying phylogeny at the starting step of Phylovar’s search algorithm (A color version of this figure appears in the online version of this article)

The second biological data consists of 16 neuron cells on which scWGS was performed to study somatic mutations in human brain development (Evrony *et al.*, 2015). Applying SCIΦ’s statistic test on the input mpileup identified 2 489 545 candidate loci. We ran Phylovar with five parallel hill-climbing chains, each for 50 000 iterations on 5 CPUs with 192 GB RAM. Phylovar finished the process after 17 h and 45 min. To compare our results with other methods, we ran Monovar and SCIΦ with default parameters. Monovar processed the data in 10 h and 26 min, while SCIΦ was still running after ten days. Phylovar’s inferred false-positive and false-negative error rates were 75.22% and 1.17%, respectively. snpEff identified 5745 non-synonymous SNVs among Phylovar’s mutation calls. Figure 6 shows hierarchical clustering on the genotypes of Phylovar, Monovar and the initial genotypes at sites with non-synonymous SNVs. We observed similarities between Monovar’s calls and the initial genotypes. By comparing the panel of Phylovar in Figure 6 with the other panels, one can see the sparse regions of mutations in panels of Monovar and the initial genotype matrix that are inferred as reference alleles by Phylovar.

Furthermore, we investigated the genes likely related to neurodegenerative diseases by comparing our findings with the genes reported in Wei *et al.* (2019), where somatic mutations were studied in 1461 control and diseased human brains with different neurodegenerative disorders. Among the genes inferred by Phylovar to harbor non-synonymous mutations, 12 genes were reported in Wei *et al.* (2019). We observed that the genes *MUC16* and *MLIP* were frequently mutated in different regions; also, non-synonymous SNVs were observed within *KRT33A* and *SEMA5B* in Wei *et al.* (2019) from patients with Creutzfeldt-Jakob and Alzheimer diseases, respectively. The presence of these non-synonymous mutations in both diseased and normal samples implies the high mutability of these genes even in a healthy individual.

## 4 Conclusions

The rapid growth of SCS technologies poses computational challenges due to the increasing number of cells and sites sequenced per genome (Lähnemann *et al.*, 2020). In this work, we focused on addressing the computational challenge associated with the breadth of genomic sites in scDNAseq data. Here, we introduced Phylovar, a scalable MLE method for phylogeny-guided inference of SNVs from single-cell DNA sequencing data suitable for scWGS and scWES data with an extensive number of loci. We introduced a novel vectorized formula for likelihood calculation, making Phylovar scalable to hundreds of thousands, even millions of loci.

We assessed Phylovar’s performance against state-of-the-art variant callers SCIΦ (Singer *et al.*, 2018) and Monovar (Zafar *et al.*, 2016), through simulated benchmarks. Phylovar outperforms SCIΦ in terms of running time while being more accurate than Monovar in different simulation scenarios. We also applied Phylovar to two real biological datasets. For a TNBC dataset with 32 single cells and 3375 candidate loci, Phylovar identified SNVs with functional impact among which 84% were supported by bulk sequencing data. Phylovar was also more accurate than Monovar and 6.5x faster than that of SCIΦ. For a larger dataset containing 16 normal human neuron cells and approximately 2.5 million candidate loci, Phylovar identified 5745 non-synonymous SNVs some of which were related to neurodegenerative diseases. Interestingly, Phylovar detected 75.22% false-positive, and 1.17% false-negative error rate for this dataset. The neuron cells data was particularly challenging due to large number of sites. For this data, SCIΦ failed to converge even after ten days of running while Phylovar terminated after less than 18 h.

Phylovar makes it possible to analyze datasets with large number of loci within reasonable time and memory requirements, thus adding to the growing toolbox for analyzing scDNAseq data. As a direction for future research, we will explore deviations from the simplified ISA model and investigate the feasibility of applying more general finite-sites models (FSM) (Zafar *et al.*, 2017, 2019) to datasets with many loci. As scDNAseq technologies advance, the sequencing cost per cell decreases (Lim *et al.*, 2020; Tang *et al.*, 2019). Consequently, we expect more scWGS and scWES datasets to emerge in the future, requiring methods such as Phylovar that can perform scalable variant calling on datasets with millions of loci.
