# Correction to: Phylovar: toward scalable phylogeny-aware inference of single-nucleotide variations from single-cell DNA sequencing data

**DOI:** 10.1093/bioinformatics/btad321

**Published:** 2023-05-23

This is a correction to: Mohammadamin Edrisi, Monica V Valecha, Sunkara B V Chowdary, Sergio Robledo, Huw A Ogilvie, David Posada, Hamim Zafar, Luay Nakhleh, Phylovar: toward scalable phylogeny-aware inference of single-nucleotide variations from single-cell DNA sequencing data, Bioinformatics, Volume 38, Issue Supplement_1, July 2022, Pages i195–i202, https://doi.org/10.1093/bioinformatics/btac254

In the originally published version of this manuscript, panels f and g of Figure 1 were duplicated.

Figure 1 has now been corrected.

